# Network-Based Transcranial Direct Current Stimulation May Modulate Gait Variability in Young Healthy Adults

**DOI:** 10.3389/fnhum.2022.877241

**Published:** 2022-06-09

**Authors:** Rong Zhou, Junhong Zhou, Yanwen Xiao, Jiawei Bi, Maria Chiara Biagi, Giulio Ruffini, Natalia A. Gouskova, Brad Manor, Yu Liu, Jiaojiao Lü, On-Yee Lo

**Affiliations:** ^1^Key Laboratory of Exercise and Health Sciences of Ministry of Education, Shanghai University of Sport, Shanghai, China; ^2^Hinda and Arthur Marcus Institute for Aging Research, Hebrew SeniorLife, Boston, MA, United States; ^3^Harvard Medical School, Harvard University, Boston, MA, United States; ^4^Neuroelectrics, Barcelona, Spain

**Keywords:** transcranial, non-invasive, direct current, network, gait, variability

## Abstract

**Purpose:**

Previous studies have linked gait variability to resting-state functional connectivity between the dorsal attention network (DAN) and the default network (DN) in the brain. The purpose of this study was to examine the effects of a novel transcranial direct current stimulation (tDCS) paradigm designed to simultaneously facilitate the excitability of the DAN and suppress the excitability of the DN (i.e., DAN+/DN-tDCS) on gait variability and other gait characteristics in young healthy adults.

**Methods:**

In this double-blinded randomized and sham-controlled study, 48 healthy adults aged 22 ± 2 years received one 20-min session of DAN+/DN-tDCS (*n* = 24) or no stimulation (the Sham group, *n* = 24). Immediately before and after stimulation, participants completed a gait assessment under three conditions: walking at self-selected speed (i.e., normal walking), walking as fast as possible (i.e., fast walking), and walking while counting backward (i.e., dual-task walking). Primary outcomes included gait stride time variability and gait stride length variability in normal walking conditions. Secondary outcomes include gait stride time and length variability in fast and dual-task conditions, and other gait metrics derived from the three walking conditions.

**Results:**

Compared to the Sham group, DAN+/DN-tDCS reduced stride length variability in normal and fast walking conditions, double-limb support time variability in fast and dual-task walking conditions, and step width variability in fast walking conditions. In contrast, DAN+/DN-tDCS did not alter average gait speed or the average value of any other gait metrics as compared to the sham group.

**Conclusion:**

In healthy young adults, a single exposure to tDCS designed to simultaneously modulate DAN and DN excitability reduced gait variability, yet did not alter gait speed or other average gait metrics, when tested just after stimulation. These results suggest that gait variability may be uniquely regulated by these spatially-distinct yet functionally-connected cortical networks. These results warrant additional research on the short- and longer-term effects of this type of network-based tDCS on the cortical control of walking in younger and older populations.

## Introduction

Gait variability refers to the degree of temporospatial fluctuations in the repetitive movement patterns that occur from stride to stride when walking. The sources that give rise to and/or regulate gait variability are not fully understood. However, evidence suggests that engaging in an attention-demanding task while walking tends to increase gait variability in healthy younger adults (Dubost et al., 2008), and relatively more so in older adults (Hausdorff et al., 2008) and in those suffering from cognitive impairment (Beauchet et al., 2017). These observations suggest that gait variability is at least in part regulated by aspects of higher-level cognitive function and attentional control (Hausdorff et al., 2005).

The magnitude of gait variability has recently been linked to the integrity of several brain regions and their connected neural networks (Lo et al., 2017; Horin et al., 2021). Lo et al. (2021) reported that in several different cohorts of older adults, the degree of gait variability during quiet walking at preferred speed was *cross-sectionally* correlated with the strength of resting-state functional connectivity between the dorsal attention network (DAN) and the default network (DN) as measured by the fluctuating blood-oxygen-level-dependent (BOLD) signals. Intriguingly, the DAN is viewed as a task-positive network (i.e., it is relatively more active during the execution of a continuous task and relatively less active during periods of rest or ‘mind-wandering’), whereas the DN is a task-negative network (i.e., it is relatively less active during execution of a continuous task and more active during rest). As such, the DAN and the DN typically function in a reciprocal fashion; that is, when the one is activated the other is suppressed (Gusnard et al., 2001; Buckner et al., 2008). These intriguing results suggest that at least under ‘normal’ walking conditions, gait variability may stem from the dynamic interplay between DAN and DN function.

Non-invasive transcranial direct current stimulation (tDCS) can safely and selectively modulate neuronal excitability by transferring low-amplitude currents between two or more surface electrodes placed upon the scalp (Nitsche and Paulus, 2011; Stagg and Nitsche, 2011). Uniquely, tDCS concurrently facilitates the excitability of one or more cortical regions in the brain via the generation of electrical current flow *into* the cortex (typically regions in close proximity to the positive ‘anode’ electrodes) and inhibits the excitability of one or more other regions (typically in close proximity to the negative ‘cathode’ electrodes). The combination of multi-electrode tDCS devices with electrical field modeling has recently afforded the development of montages (i.e., electrode placement and current flow parameters) that create more focal electric fields, and thus, enable researchers to better control simultaneous excitatory and inhibitory effects (Ruffini et al., 2014; Fischer et al., 2017; Mencarelli et al., 2020).

The aim of this pilot randomized and double-blinded study was to provide experimental evidence for a causal role of brain network function in the regulation of gait variability. To do so, we utilized advanced tDCS technology to develop a novel form of tDCS designed to concurrently facilitate the excitability of primary nodes of the DAN and inhibit the excitability of primary nodes of the DN. We hypothesized that a single exposure of this type of tDCS, as compared to sham stimulation, would reduce gait variability in healthy young adults, when tested just after stimulation.

## Materials and Methods

### Participants

Based upon pilot tests (Wrightson et al., 2015), we calculated that a sample size of 48 participants would provide 87% power (at a two-tailed alpha level of 0.05) for detecting differences in gait variability between groups at an effect size of 0.4. Participant inclusion criteria were: (1) right-handed as determined by the Edinburgh Handedness Inventory (Oldfield, 1971) and (2) the ability to walk for 60 s without personal assistance. Exclusion criteria were: (1) self-reported pain in the legs or feet or other chronic lower-extremity medical issues significantly affecting gait, (2) hospitalization within the past 6 months for any reason, (3) the use of neuro-active drugs that may impact brain state, (4) self-reported cerebral or cardiovascular diseases, neurological diseases (e.g., Parkinson’s disease, stroke, etc.), or musculoskeletal disorders known to affect gait, (5) cognitive dysfunction as defined by Mini-Mental Status Exam < 24 (Folstein et al., 1975), and (6) any contraindications with respect to the use of tDCS (e.g., metal-implanted devices in the brain).

All study participants provided written informed consent as approved by the institutional review board of the Shanghai University of Sports (102772020RT109), prior to screening and all other study procedures.

### Experimental Protocol

A double-blinded, randomized, and sham-controlled study was completed in which participants were randomly assigned to one of two groups: the DAN+/DN-tDCS group or the Sham group. Each participant completed one visit consisting of a comprehensive gait assessment immediately before and after receiving 20 continuous minutes of either DAN+/DN-tDCS or no stimulation. Participants were asked not to exercise vigorously in the 24 h prior to the test and not to drink any beverages containing stimulants such as caffeine for 4 h prior to the test (Zulkifly et al., 2020).

### Transcranial Direct Current Stimulation

**FIGURE 1:**
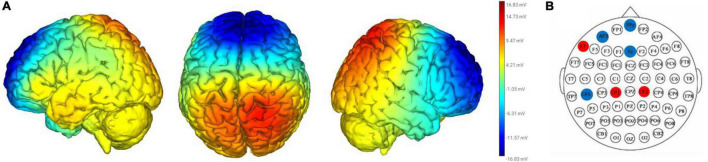
Electrical current flow model **(A)** and electrical placements **(B)** of the DAN+/DN-tDCS montage. The heat map in **(A)** reflects the strength and polarity of the electrical current flow modal. Red and blue represents positive and negative electrical currents. Darker and lighter colors mean stronger and weaker electrical currents. The red and blue circles in **(B)** depicts placement of anodal electrodes (red circles: F7, CP1, and CP2) and cathodal electrodes (blue circles: FPz, AF3, Fz, and CP5), which were placed according to the 10–20 electrode placement system and held in place with a custom Neoprene^®^ cap with prefabricated holes.

The DAN+/DN-tDCS montage (i.e., electrode positions and delivered currents) was determined by the Stimweaver^®^ optimization algorithm (Ruffini et al., 2014; Fischer et al., 2017). The targeted electrical field (En^Target^) for the active montage was developed to simultaneously excite the DAN and inhibit the DN. The targeted DAN and DN regions were mapped based on the fMRI (functional Magnetic Resonance Imaging) binary volumetric images. The En^Target^ was assigned as +0.50, −0.50, and 0 V/m toward the areas of the DAN, DN, and the rest of the cortex. The modeling of the DAN+/DN-tDCS resulted in the placements of electrodes on the AF3, CP1, CP2, CP5, F7, FPZ, and FZ, of the 10–10 EEG placement system (Figure 1). The total maximum total injected current was 4.0 mA.

The DAN+/DN- tDCS was delivered with the participant seated and resting for 20 min, including a 30-s ramp-up and a 30-s ramp-down period. This approach was selected for this study because evidence suggests that anodal tDCS delivered at rest modulates cortical excitability for up to 90 min thereafter (Nitsche and Paulus, 2001) and longer durations (e.g., >25 min) of stimulation may have unintended effects (Vignaud et al., 2018; Hassanzahraee et al., 2020). Our goal for this study was to determine if gait performance was altered during this after-effect phase.

Sham group utilized the same montage yet stimulation was only delivered during the initial 30-s ramp-up and the final 30-s ramp-down periods. This approach has been demonstrated to induce similar cutaneous sensations as tDCS yet not significantly alter the excitability of cortical tissue (Gandiga et al., 2006). The group assignment was coded and selected for each participant by study personnel uninvolved in any other procedures of this study, such that neither the participants nor the study personnel conducting assessments was aware of group assignment. Blinding efficacy was assessed by asking participants to state if they believed they received tDCS or the Sham at the end of their visit. The incidence and severity of tDCS-related side effects were assessed with a short questionnaire (Brunoni et al., 2011).

### Gait Assessment

Gait assessments were completed over a 16-foot GAITRite pressure mat (ProtoKinetics Zeno Walkway, ZenoMetrics, LLC, Peekskill, NY, United States, 120 Hz sampling frequency). Each participant completed three trials of walking over the mat in each of the following conditions: (1) walking quietly at a self-selected, comfortable speed (i.e., normal walking), (2) walking as fast as possible without running (i.e., fast walking), and (3) walking at a self-selected, comfortable speed while performing verbalized serial subtractions (i.e., dual-task walking). The DAN+/DN-tDCS was developed based on the previous discovery that the magnitude of gait variability present when walking quietly at preferred speed was correlated with the strength of functional connectivity between the DAN and DN (Lo et al., 2021). We thus designated stride length and stride time variability derived from this walking condition as the primary study outcomes. Secondarily, we also examined other aspects of gait during both fast walking and dual-task walking in order to more fully examine the effects of stimulation on locomotor control.

The order of these trials was randomized, and 60 s of the break were provided between trials. Participants began each trial standing four meters away from the gait mat. They were instructed to walk straightforward to pass over the gait mat, turn 180 degrees, and walk again over the mat and back to the starting position. Participants completed this ‘lap’ three times per trial. For the dual-task condition, participants were asked to walk at their self-selected speed while performing verbalized serial subtractions of 7 from a random, three-digit number between 799 and 999 that was provided just prior to the start of the trial (Zhou et al., 2014). No instructions on task priority were given.

Gait characteristics of each trial were captured by the pressure mat. We first calculated the length, time, speed (i.e., the ratio of the length to time), step width (i.e., the perpendicular distance between the line connecting the two ipsilateral foot heel contacts with the contralateral heel contact), and double support time (i.e., the period when both feet are in contact with the ground during stance phase) for each stride. Each stride was determined from the heel strike of one foot to the following heel strike of the same foot. The mean and coefficient of variation (i.e., the ratio of the standard deviation to the mean) were then calculated for each above-mentioned gait outcome. The trial mean and coefficient of variation for each outcome were then averaged across the three walking trials that were completed for each walking condition.

### Study Outcomes

Designated primary outcomes were stride length variability and stride time variability during normal walking, as these are the two most commonly used gait outcomes (Gabell and Nayak, 1984; Beauchet et al., 2005). Secondary outcomes included stride length variability and stride time variability in the fast and dual-task walking conditions, as well as other metrics of gait variability (gait speed variability, step-width variability, and double support time variability) and the mean values for each of the above gait outcomes in each of the three walking conditions.

### Statistical Analysis

Baseline characteristics of the participants were compared across groups using two-sample *t*-tests when the variable was normally distributed. Mann–Whitney *U* tests were used for those variables that were not normally distributed.

The effect of DAN+/DN-tDCS on primary and secondary outcomes was examined by Analysis of Covariance (ANCOVA). The dependent variable for each model was each of the primary or secondary post-stimulation outcomes and the independent variable was group (i.e., DAN+/DN-tDCS, Sham), adjusting for its own pre-stimulation outcome. The Shapiro–Wilk test was used to examine the normality of the outcomes. Non-normally distributed outcomes were log-transformed prior to fitting the ANCOVA model.

The effects of the stimulation group on blinding efficacy and tDCS-related side effects were examined using Chi-Square Tests, or the Fisher’s Exact Test if the expected cell frequencies were less than five. The significance level for the primary outcomes was set to *p* < 0.025, adjusted for multiple comparisons. The significance level was set to *p* < 0.05 for all other outcomes.

For blinding efficacy, if the Chi-Square or Fisher’s Exact Test revealed a significant *p*-value, we conducted additional sensitivity analyses by adding participant blinding responses to the ANCOVA model to further examine whether the effects of stimulation on gait were influenced by one’s belief in the type of stimulation they received. JMP software version 16 (SAS Institute, Cary, NC, United States) was used for all analyses.

## Results

All 48 participants completed the entire study protocol. Group characteristics are provided in Table 1. All outcomes listed in Table 1 were normally distributed and there were no significant differences between groups (*t* < 1.08, *p* > 0.29). Gait characteristics at baseline (i.e., before tDCS or Sham) were also similar between the tDCS and Sham groups (0.006 < *t* < 1.57, 0.12 < *p* < 0.95).

**TABLE 1:** Basic information of the participants.

Variables	DAN+/DN-tDCS (*n* = 24)	Sham (*n* = 24)	*t*	*p*
Sex [*n* (%) = females]	12(50%)	12(50%)	−	1.000
Age (years)	22.83 ± 2.26	22.13 ± 2.29	1.079	0.286
Height (cm)	170.50 ± 8.88	171.92 ± 10.73	–0.498	0.621
Weight (kg)	63.42 ± 13.48	64.50 ± 12.65	–0.287	0.775

### The Effects of DAN+/DN-tDCS on Gait Characteristics

**FIGURE 2:**
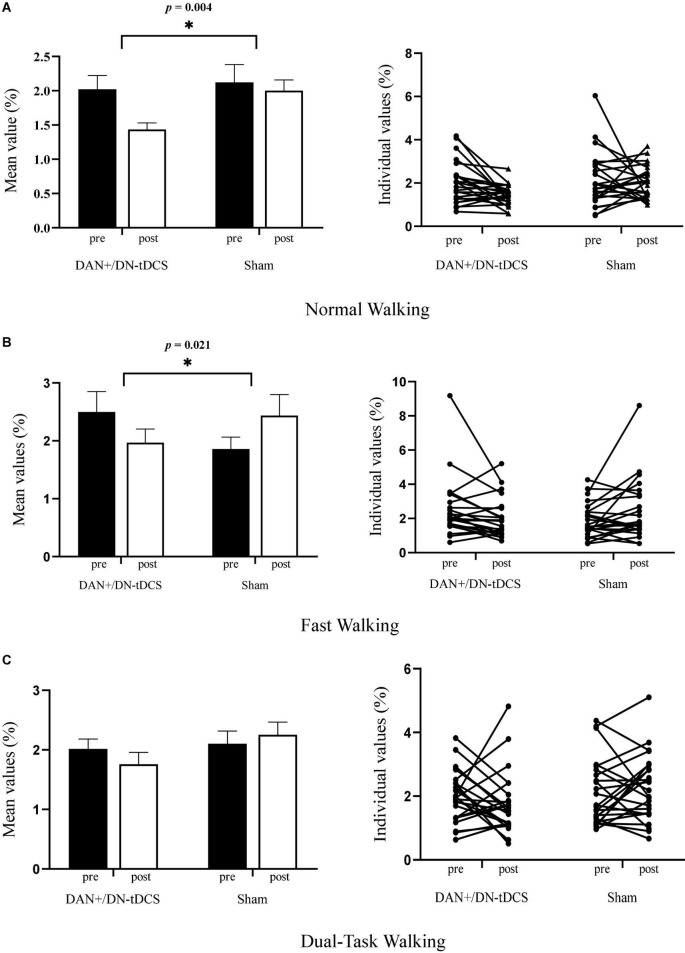
The effects of DAN+/DN-tDCS and Sham stimulation on gait stride length variability* for group mean values (left column, with the error bars representing the standard errors) and individual values (right column) in three walking conditions. **(A)** Normal walking condition: compared to Sham stimulation, DAN+/DN- tDCS induced greater reduction in stride length variability (*p* = 0.004). Individually, 17 out of 24 exhibited reduced gait variability after the DAN+/DN-tDCS, whereas only 10 out of 24 had reduced gait variability after Sham stimulation. **(B)** Fast walking condition: Compared to Sham stimulation, DAN+/DN- tDCS led to greater reduction in stride length variability (*p* = 0.021). Individually, 18 out of 24 exhibited reduced gait variability after the DAN+/DN-tDCS, whereas 10 out of 24 had reduced gait variability after Sham stimulation. **(C)** Dual-task walking condition: Compared to Sham stimulation, DAN+/DN- tDCS induced a non-significant trend toward a reduction in stride length variability (*p* = 0.07). Individually, 15 out of 24 exhibited reduced gait variability following DAN+/DN-tDCS, whereas 10 out of 24 had reduced gait variability after the Sham stimulation. *Gait stride length variability was defined as the coefficient of variation (CoV) of stride length (%).

ANCOVA analysis revealed a significant group effect for stride length variability in the normal walking (*F* = 9.50, *p* = 0.004, $\eta_{\text{p}}^{2}$ = 0.17) and fast walking (*F* = 5.69, *p* = 0.021, $\eta_{\text{p}}^{2}$ = 0.11) conditions (Figure 2), double-limb support time variability in the fast walking (*F* = 5.03, *p* = 0.033, $\eta_{\text{p}}^{2}$ 0.10) and dual-task walking (*F* = 5.38, *p* = 0.025, $\eta_{\text{p}}^{2}$ = 0.11) conditions, and step width variability in the dual-task walking condition (*F* = 4.15, *p* = 0.048, $\eta_{\text{p}}^{2}$ = 0.084) (Table 2). In all cases, after adjusting for their pre-stimulation value, the magnitude of gait variability reduced more following DAN+/DN-tDCS, as compared to that of the Sham group. In particular, following tDCS, stride length variability reduced 29.2 and 21.2% in the normal and fast walking conditions. Double support time variability reduced 36.9 and 29.0% in the fast and dual-task walking conditions, and step width variability reduced 20.7% in dual-task walking. In contrast, each of the above outcomes reduced slightly or even appeared to increase from pre-to-post in the Sham group (Table 2).

**TABLE 2:** Effects of DAN+/DN-tDCS and Sham group on various characteristics of gait variability.

Coefficient of variance (%, mean ± *SD*)		DAN+/DN-tDCS		Sham		Group effect
		Pre	Post	Pre	Post	*p*
Gait stride length variability	Normal walking	2.02 ± 0.97	1.43 ± 0.47	2.12 ± 1.27	2.00 ± 0.76	0.004*
	Fast walking	2.50 ± 1.73	1.97 ± 1.15	1.86 ± 1.00	2.44 ± 1.78	0.021*
	Dual-task walking	2.02 ± 0.81	1.76 ± 0.98	2.10 ± 1.04	2.25 ± 1.05	0.072
Gait stride time variability	Normal walking	2.16 ± 0.89	1.79 ± 0.62	2.13 ± 0.92	1.94 ± 0.97	0.629
	Fast walking	2.15 ± 1.07	1.89 ± 1.06	2.14 ± 0.98	2.02 ± 1.11	0.515
	Dual-task walking	2.38 ± 0.90	1.80 ± 0.76	2.32 ± 0.95	2.20 ± 1.02	0.396
Gait speed variability	Normal walking	2.95 ± 1.15	2.37 ± 0.84	2.71 ± 1.08	2.62 ± 0.67	0.200
	Fast walking	3.20 ± 1.19	2.72 ± 1.22	2.81 ± 1.31	2.91 ± 1.61	0.353
	Dual-task walking	3.18 ± 1.22	2.73 ± 1.21	3.05 ± 1.20	3.04 ± 1.51	0.289
Gait step width variability	Normal walking	23.20 ± 8.69	21.14 ± 8.13	22.72 ± 6.35	24.25 ± 8.23	0.387
	Fast walking	23.57 ± 9.81	19.34 ± 7.48	23.91 ± 8.75	22.57 ± 8.09	0.147
	Dual-task walking	26.07 ± 13.93	20.68 ± 6.26	22.22 ± 8.74	25.86 ± 14.25	0.048*
Double-limb support time variability	Normal walking	4.17 ± 1.52	3.44 ± 1.11	4.68 ± 2.74	4.62 ± 2.76	0.614
	Fast walking	6.25 ± 3.37	4.71 ± 1.88	6.16 ± 3.49	5.94 ± 2.14	0.033*
	Dual-task walking	5.28 ± 2.21	3.75 ± 1.29	5.08 ± 1.66	5.30 ± 3.18	0.025*

*Significant differences for post stimulation outcomes between the two groups from the ANCOVA model (p < 0.025 for primary outcomes and p < 0.05 for all the other outcomes).

On the other hand, average gait speed and the mean value of all other gait metrics (Table 3) were not significantly different after DAN+/DN-tDCS, as compared to the Sham group (*p* > 0.267).

**TABLE 3:** Effects of DAN+/DN-tDCS and Sham group on mean values-based gait parameters.

Mean values-based outcomes (mean ± *SD*)		DAN+/DN-tDCS		Sham		Group effect
		Pre	Post	Pre	Post	*p*
Gait stride length (cm)	Normal walking	139.15 ± 12.77	141.29 ± 14.67	142.02 ± 12.78	142.41 ± 13.24	0.412
	Fast walking	163.09 ± 14.96	161.22 ± 15.26	169.15 ± 19.76	165.80 ± 18.84	0.493
	Dual-task walking	133.55 ± 13.48	133.60 ± 13.16	135.27 ± 16.32	134.49 ± 17.29	0.676
Gait stride time (sec)	Normal walking	1.09 ± 0.07	1.09 ± 0.07	1.10 ± 0.05	1.11 ± 0.08	0.695
	Fast walking	2.15 ± 1.07	1.89 ± 1.06	2.14 ± 0.98	2.02 ± 1.11	0.764
	Dual-task walking	2.38 ± 0.90	1.80 ± 0.76	2.32 ± 0.95	2.20 ± 1.02	0.396
Gait speed (cm/sec)	Normal walking	128.14 ± 14.49	131.13 ± 19.07	129.79 ± 14.34	129.16 ± 16.15	0.267
	Fast walking	179.79 ± 20.51	174.56 ± 15.91	181.12 ± 24.52	176.73 ± 22.82	0.696
	Dual-task walking	119.92 ± 14.25	120.45 ± 13.93	120.26 ± 20.70	117.72 ± 23.23	0.320
Gait step width (cm)	Normal walking	9.46 ± 2.99	9.43 ± 2.71	9.15 ± 2.45	8.83 ± 2.32	0.387
	Fast walking	9.49 ± 3.02	9.55 ± 2.82	9.11 ± 2.32	9.09 ± 2.79	0.767
	Dual-task walking	9.35 ± 3.18	9.47 ± 2.53	9.06 ± 2.42	8.92 ± 2.59	0.424
Double-limb support time (sec)	Normal walking	0.29 ± 0.04	0.29 ± 0.04	0.30 ± 0.04	0.30 ± 0.04	0.614
	Fast walking	0.20 ± 0.04	0.21 ± 0.03	0.21 ± 0.03	0.21 ± 0.03	0.348
	Dual-task walking	0.31 ± 0.04	0.32 ± 0.03	0.33 ± 0.06	0.34 ± 0.08	0.344

### The Effects of DAN+/DN- tDCS on Subtraction Task During Dual-Task Walking

The percentage of correct answers for a given responses of the subtraction task during dual-task walking was high in both groups and unaffected by stimulation (tDCS: pre: 93.7 ± 6.4%, post: 93.97 ± 9.55%; Sham: pre: 92.83 ± 6.6%, post: 90.90 ± 10.91%).

### Blinding Effects

Sixteen (67%) participants in the DAN+/DN-tDCS group and seven (29%) participants in the Sham group guessed that they received real tDCS. The Chi-Square test of blinding responses indicated a significant effect of the stimulation group (*p* = 0.03). The DAN+/DN-tDCS group was also more likely to self-report tingling (*p* = 0.01), itching (*p* = 0.04), pain (*p* = 0.04), and redness (*p* = 0.002) (Table 4).

**TABLE 4:** Number of self-reported side-effect events of DAN+/DN-tDCS and Sham groups.

	DAN+/DN-tDCS	Sham	*p*-value
Tingling*	20/24 = 83.3%	11/24 = 45.8%	0.01*
Itching*	17/24 = 70.8%	10/24 = 41.7%	0.04*
Pain*	16/24 = 66.7%	8/24 = 33.3%	0.04*
Redness*	11/24 = 45.8%	1/24 = 4.2%	0.002*
Burning	10/24 = 41.7%	5/24 = 20.8%	0.2
Fatigue	4/24 = 16.7%	5/24 = 20.8%	1.00

The *sign indicates a significant difference between two groups.

These results of blinding efficacy and side effects suggested that the employed sham protocol served as a sub-optimal control for real stimulation. We therefore added participant guesses of the type of stimulation they received into the ANCOVA model to examine whether the observed effects of stimulation on gait variability were influenced by this belief. All previously reported significant group differences in gait outcomes remained significant, suggesting that the effects of DAN+/DN-tDCS were not driven entirely by placebo effects.

## Discussion

The results of this study indicate that tDCS designed to simultaneously facilitate the excitability of the DAN and suppress the excitability of the DN appears to alter the regulation of gait variability in young adults. Specifically, while tDCS as compared to the Sham group reduced numerous aspects of gait variability across three different walking task conditions, this novel form of non-invasive brain stimulation did not affect gait speed or other average gait characteristics. These results suggest that gait variability may be uniquely regulated by the function of one or both of these two spatially-distinct brain networks.

Previous research has indicated that gait variability appears to be linked to the structure and function of several brain regions located within the DAN or the DN, as well as the functional connectivity between these two networks (Lo et al., 2017, 2021). The primary function of the DAN is to orient one’s attention toward external targets and goal-oriented tasks (Corbetta and Shulman, 2002), while the DN is dedicated to mind-wandering, self-reflection, and conceptual processing (Fransson, 2005). These two brain networks are typically activated and deactivated in a negative, reciprocal manner; that is when the DAN is activated the DAN is suppressed, and vice versa. Functionally, this negative correlation between DAN and DN is believed to subserve sustained attention defined as the ability to remain persistent and devote continuous effort over extended periods of time (Esterman et al., 2013; Fortenbaugh et al., 2018). Along these lines, walking is a continuous task that appears to also require sustained attention (and several additional aspects of cognitive function) for stable performance over time (Dixon et al., 2017, 2018; Wang et al., 2019). The observation that gait variability was altered following tDCS targeting the DAN and DN provides unique, causal evidence implicating a likely role of one or both of these networks in the regulation of gait variability in healthy younger adults.

Interestingly, while a non-significant trend toward reduced gait variability in the dual task condition was observed following tDCS, the effects of stimulation were noticeably less in this walking condition as compared to the others. We expect that this is because dual task performance likely depends upon still other cognitive networks (e.g., the fronto-parietal executive network) and the ability to effectively allocate ‘resources’ between the two tasks (Lo et al., 2021; Maidan et al., 2022). On the other hand, it might also be that higher intensity or longer stimulation is needed to induce changes in cortical function that are sufficient to cause measurable differences in gait within more challenging conditions.

Our results suggested the DAN+/DN-tDCS, when compared to Sham stimulation, significantly influenced gait performance. While the total current for tDCS was 4mA, stimulation was designed to simultaneously target spatially-distinct regions of the brain. While this approach is aligned with previous research suggesting that 4 mA direct current is safe and tolerable in younger (Nitsche and Bikson, 2017; Khadka et al., 2020) and older (Manor et al., 2021; Zhou et al., 2021) adults, the effects of the tested montage on cortical excitability and other aspects of neuronal function were not examined. There also exists the possibility that the tested Sham control either directly or indirectly modulated cortical excitability by a non-trivial amount. Future studies incorporating fMRI, EEG, and or TMS are thus warranted to study the effects of these forms of stimulation on cortical function and its relationship to gait performance in younger and older populations.

The impact of tDCS on the brain appears to be dependent upon the state of the participant’s brain during stimulation (Sriraman et al., 2014; Cabral et al., 2015). Thus, while the current study indicated that tDCS delivered with the brain in a resting state improves gait performance when tested just after stimulation, future efforts that deliver tDCS while the participant is walking or performing other tasks may alter and potentially improve its short and relatively longer-term effectiveness.

This pilot study had several limitations. First, we did not use neurophysiological assessments to study the effects of the tested tDCS montages on cortical function. The included model (Figure 1) was only intended to illustrate the strength and polarity of the electric field generated by the tested tDCS montage on a standard brain. This model therefore does not necessarily reflect the specific characteristics of the generated electric field that are believed to drive changes in neuronal excitability; for example, the *E*-field component normal to the cortical surface (Ruffini et al., 2014). Moreover, the *E*-field generated by tDCS for each participant is likely to vary from the shown model due to individual variance in head and brain anatomy. Future work is needed to (1) understand the effects of this type of tDCS on large-scale brain network excitability and function and (2) the extent to which such effects are influenced by inter-individual variation in head and brain anatomy. In addition to the inter-individual differences in brain structure and organization, there may be other potential sources to contribute to the variable responses such as sex and genetics. Future work should attempt to delineate the sources of this inter-individual variance in the effects of stimulation.

The implemented inactive sham protocol provided suboptimal masking of stimulation. Thus, while results were not influenced by subjective guess of the type of stimulation received, future efforts should consider using active sham approaches, which appear to improve the blinding of both participants and research staff (Zhou et al., 2021). Moreover, as we only designed and tested the DAN+/DN-tDCS montage for this study, future efforts examining the effects of the same montage with reversed direction of current flow (i.e., a DAN-/DN+) would help to determine if the observed effects on gait were a result of a general effect of stimulation on brain function, or the more specific result of increasing the excitability of DAN and decreasing the excitability of the DN. Future work is also warranted to examine the effects of DAN+/DN-tDCS on tests of sustained attention, such as the gradual onset continuous performance test (gradCPT) (Esterman et al., 2013) or the Eriksen flanker task (Kelly et al., 2008), in order to discern whether observed effects on gait variability are related to measurable changes in sustained attention or other aspects of cognitive function. Lastly, the observation that DAN+/DN-tDCS reduced gait variability in younger adults warrants additional research to determine the acute- and longer-term effects of this form of non-invasive brain stimulation in older adults and other clinical populations with elevated gait variability.

## Conclusion

This study demonstrates that a single exposure to tDCS designed to simultaneously modulate DAN and DN excitability reduced gait variability, yet did not alter gait speed or other average gait metrics in healthy young adults, when tested just after stimulation. Although there is no imaging evidence, these results of ours suggest that gait variability may be uniquely modulated by these spatially distinct but functionally linked cortical networks. Future research is needed to further investigate the short- and long-term effects of this network-based tDCS on the cortical control of walking in both young and older populations, as well as to explore its neural mechanisms and to increase the evidence of its effectiveness.

## Data Availability Statement

The original contributions presented in this study are included in the article/supplementary material, further inquiries can be directed to the corresponding authors.

## Ethics Statement

The studies involving human participants were reviewed and approved by the Institutional Review Board of the Shanghai University of Sports (102772020RT109). The patients/participants provided their written informed consent to participate in this study.

## Author Contributions

O-YL, MB, and GR developed this tDCS montage. RZ, JZ, YL, JL, and O-YL contributed to the conception and design of the study. RZ, YX, and JB helped with data collection. RZ and JZ drafted the manuscript. O-YL and BM provided critical revision of the manuscript. YL, JL, NG, and O-YL conducted the statistical analyses and provided data interpretation. All authors read, edited, and approved the final version of the manuscript.

## Conflict of Interest

The authors declare that the research was conducted in the absence of any commercial or financial relationships that could be construed as a potential conflict of interest.

## Publisher’s Note

All claims expressed in this article are solely those of the authors and do not necessarily represent those of their affiliated organizations, or those of the publisher, the editors and the reviewers. Any product that may be evaluated in this article, or claim that may be made by its manufacturer, is not guaranteed or endorsed by the publisher.

## Abbreviations

DAN: dorsal attention network
DN: the default network
tDCS: transcranial direct current stimulation
DAN+/DN-tDCS: simultaneously facilitate the excitability of the DAN and suppress the excitability of the DN.
